# Evaluating the accuracy and increasing the reliable diagnosis rate of blood tests for liver fibrosis in chronic hepatitis C

**DOI:** 10.1111/j.1478-3231.2008.01789.x

**Published:** 2008-11

**Authors:** Paul Calès, Victor de Ledinghen, Philippe Halfon, Yannick Bacq, Vincent Leroy, Jérôme Boursier, Juliette Foucher, Marc Bourlière, Anne de Muret, Nathalie Sturm, Gilles Hunault, Frédéric Oberti

**Affiliations:** 1 Service d'Hépato-gastroentérologie et laboratoire HIFIHIFR 132, CHU, Angers, France; 2 Service d'Hépato-gastroentérologie, Hôpital du Haut LévèquePessac, CHU Bordeaux, France; 3 Laboratoire AlphabioMarseille, France; 4 Service d'Hépato-gastroentérologie, Hôpital TrousseauCHRU, Tours, France; 5 Clinique d'Hépato-gastroentérologie, pôle digestif-DUNE, CHU, & INSERM/UJF U823IAPC, IAB, Grenoble, France; 6 Service d'Hépato-gastroentérologie, Hôpital Saint-JosephMarseille, France; 7 Service d'Anatomopathologie, Hôpital Trousseau, CHRUTours, France; 8 Service d'Anatomopathologie, CHUGrenoble, France

**Keywords:** blood test, diagnostic accuracy, liver biopsy, liver fibrosis, Metavir staging, non-invasive diagnosis, reliable diagnosis, sensitivity, specificity, viral hepatitis C

## Abstract

**Background:**

The reliable diagnosis rate of diagnostic tests is provided by their intervals with acceptable accuracy (e.g. ≥90%) where a liver biopsy can be avoided.

**Aims:**

To evaluate the overall accuracy and improve the reliable diagnosis rates of blood tests for significant liver fibrosis.

**Methods:**

Five blood tests were compared with Metavir fibrosis (F) staging in 1056 patients with chronic hepatitis C.

**Results:**

Area under the receiver operating characteristics (F0-1 vs. F2-4) were: FibroMeter: 0.853, Fibrotest: 0.811, Fib-4: 0.799, aspartate aminotransferase to platelet ratio index (APRI): 0.786 and Hepascore: 0.784 (*P*<10^−3^ between tests). The reliable diagnosis rates based on two traditional intervals defined by thresholds ≥90% of negative predictive values (NPV) and positive predictive values (PPV), diagnosing F0/1 and F2/3/4, respectively, were: FibroMeter: 43.5%, APRI: 19.6%, Fibrotest: 17.1%, Hepascore: 3.9%, Fib-4: 1.7% (*P*<10^−3^). By dividing the indeterminate interval by the diagnostic cut-off, two new intervals could be diagnosed reliably: F1/2 and F1/2/3. Accordingly, the reliable diagnosis rate was increased, e.g. FibroMeter: 75.5% (accuracy: 89.5%) with three intervals (F0/1, F1/2, F2/3/4). It was possible to further increase this rate by using the more exportable 90% sensitivity/specificity thresholds, e.g. FibroMeter: 90.2% (accuracy: 86.4%). By using the four intervals, the reliable diagnosis rate was 100% (accuracy: 89.5% with predictive value (PV) and 87.5% with sensitivity/specificity).

**Conclusion:**

Reliable diagnosis is a diagnostic index devoted to clinical practice. Its rate can be increased by creating new intervals between diagnostic cut-off and 90% PVs or sensitivity/specificity thresholds. This increased the overall accuracy from 78.1 to 89.5% and reduced the need for a liver biopsy from 56.5 to 0% with the most accurate test.

Score-based blood tests for liver fibrosis that take into account several blood or clinical markers have been available for about 10 years (1). They are usually designed to diagnose significant fibrosis, which includes all stages with bridging fibrosis (2). Significant fibrosis has been adopted as a target because it can be identified using semiquantitative histological staging in a patient with chronic hepatitis C and because it is an indicator for treatment. The overall accuracy of several blood tests is considered to be excellent. However, this accuracy is variable as a function of blood test value with maximum accuracy at the extreme values and minimum accuracy in the median values (see Fig. 1). A reliable diagnosis corresponds to the intervals of blood test values where the diagnostic accuracy is considered to be sufficiently reliable for clinical practice. Thus, in these patients, a liver biopsy is considered to be avoidable. Previously, the intervals of reliable diagnosis were defined by the thresholds provided by 90% predictive values (PV) (3, 4). However, how these intervals are determined has never been examined or commented on in detail and thorough statistical consideration might cast some doubts on the previous statements.

**Fig. 1:**
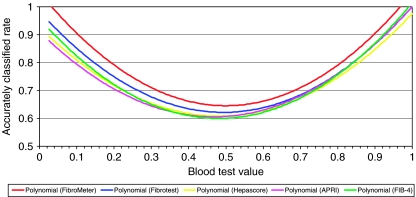
Diagnostic accuracy as a function of the value of five blood tests (split into 2.5% percentiles). Curves were smoothed by polynomial regression.

Thus, the main aim of this study was to describe comprehensively the performance of original blood tests in identifying intervals of reliable diagnosis, and to examine whether it was possible to improve reliable diagnosis rates for significant fibrosis. The secondary aims were to compare the overall accuracy and reliable diagnosis rates between several blood tests and evaluate other diagnostic targets. To achieve these objectives, we performed a meta-analysis with individual data using a large population of patients for whom liver biopsy and numerous blood markers were available for evaluation and comparison. We decided to compare two simple tests (based on ratio), aspartate aminotransferase to platelet ratio index (APRI) (5) and Fib-4 (6), including indirect fibrosis markers, and three sophisticated tests, based on algorithms provided by logistic regression: Fibrotest including indirect markers (3) and two mixed tests, including indirect and direct markers: Hepascore (7) and FibroMeter (8).

## Methods

### Data source

Using the Medline database and a manual search, we systematically reviewed the literature from 1997 to June 2007 for studies comparing FibroMeter and Fibrotest in patients with chronic viral hepatitis C for whom liver biopsy data were available. Information provided by the tests enabled Hepascore, APRI and Fib-4 to be calculated. Three independent publications were retrieved. The first study, involving one centre, Angers, was the original publication that described the FibroMeter test (8). The second study included two independent centres, Provence/Côte d'Azur (PACA) and Tours (9), and the third was from Grenoble (4). The PACA centre incorporated three secondary and two tertiary care settings. Two additional, currently unpublished populations were provided by Angers and Bordeaux (10). Thus, individual patient data were available from five centres, independent for study design, patient recruitment, blood marker determination and interpretation of liver histology.

### Patients

The inclusion and exclusion criteria were very similar at all five centres. Patients with chronic viral hepatitis C were prospectively included from 1994 to 2007 if they had anti-hepatitis C virus (HCV) antibodies, HCV RNA in serum and available liver biopsy and blood markers. Fasting blood samples were collected immediately before or no more than 3 months after the liver biopsy was performed. Patients in the Tours and PACA centres were excluded if their liver specimens were smaller than 15 mm. Other exclusion criteria were additional causes of liver disease, particularly HIV or hepatitis B virus co-infection, complicated cirrhosis, anti-fibrotic treatment in the previous 6 months and alcohol consumption of more than 30 g/day in the 5 years before inclusion. Overall, the five centres provided 1535 patients; 479 were not included because of missing criteria or data, leaving a core population of 1056 patients. The study protocol conformed to the ethical guidelines of the current Declaration of Helsinki and was approved by local ethics committees.

### Blood measurements

Blood samples were processed independently at each centre. The variables determined were platelet count, urea, bilirubin, γ-glutamyl transpeptidase, aspartate aminotransferase (AST) and alanine aminotransferase, prothrombin index, apolipoprotein A1, haptoglobin, hyaluronic acid (Corgenix) and α-2-macroglobulin (Dade Behring). Direct markers were measured in either fresh blood or frozen samples of serum stored at ≤−20 °C. Indirect markers were usually measured in fresh blood. Automats and assay techniques varied between the centres (details not provided), with the exception of apolipoprotein, α-2-macroglobulin and hyaluronic acid. Blood tests were calculated according to published formulas (5–8, 10). The FibroMeter score was slightly improved by taking patient sex into account as described in a previous study (11). AST used in APRI was divided by a common upper limit of normal, as several studies performed in numerous laboratories have shown excellent inter-laboratory reproducibility (12).

### Liver biopsy

Liver biopsies were performed using Menghini's technique with a 1.4–1.6-mm-diameter needle. Biopsy specimens were fixed in a formalin–alcohol–acetic solution and embedded in paraffin; 5-μm-thick sections were then cut and stained with haematoxylin–eosin–saffron. Liver fibrosis was staged from F0 to F4 according to the Metavir staging system (13). Three diagnostic targets were defined as follows: significant fibrosis (main target), F2+F3+F4; severe fibrosis, F3+F4; and cirrhosis, F4. Readings were performed by independent blinded senior pathologists specialized in hepatology. Histological assessments were performed twice by the same pathologist in Grenoble, once in Bordeaux and once by each of two pathologists in Angers, Tours and PACA, with a common final reading in cases of disagreement. Biopsy specimens were not re-examined centrally, as we have shown in a previous study (involving most of the same pathologists) that inter-observer agreement on the Metavir staging system is excellent among senior hepato-pathologists (14).

### Methodology

#### End points

The main objective of this study was to evaluate the rates of overall accuracy and reliable diagnosis of blood tests for significant fibrosis and other ways to increase the rate of reliable diagnosis. Secondary objectives were to evaluate the overall accuracy for other diagnostic targets, to compare the performance of blood tests and to evaluate the robustness of PVs.

#### Definitions

##### Diagnostic target

The main objective of a blood test, usually defined by two ranges of all stages of fibrosis, with a *diagnostic cut-off* between them, e.g. significant fibrosis (yes/no).

##### Overall accuracy (or diagnostic accuracy or correct diagnosis)

Sum of true positives and negatives as a proportion of the whole population.

##### Reliable diagnosis

Corresponds to the intervals of blood test values where the diagnostic accuracy is considered to be sufficiently reliable for clinical practice. Considering the diagnostic target of blood test in clinical practice, this means that a liver biopsy can be avoided. Thus, with the traditional definition based on 90% PVs, a reliable diagnosis for a patient means ≥90% chance to be F0/1 in the lower interval and ≥90% chance to be ≥F2 in the highest interval of blood test values. The indeterminate interval is the zone outside of these reliable intervals.

##### Diagnostic cut-off

The diagnostic cut-off of a blood test value, usually provided by binary logistic regression, distinguishes patients with or without the diagnostic target. Here, it was fixed in two ways: *a priori* to 0.5 according to statistical convention (with the exception of APRI and Fib-4, which are simple ratios), and *a posteriori* according to the highest Youden index (Se+Spe−1) or the maximum overall accuracy to optimize test performance. The fixed *a priori* cut-off does not always provide the highest performance, particularly when it is applied in populations larger than the original one.

##### Classification/misclassification rate

This method was called ‘performance profile’ in a recent article (9). Briefly, the misclassification rate of a blood test for significant fibrosis was calculated using Metavir staging as a reference. Thus, a patient with Metavir F0 or F1 classified in the significant fibrosis group by blood test was considered as misclassified and *vice versa*. The misclassification rate was calculated in each, or in possibly combined Metavir fibrosis (F) stage(s) as determined by histological staging. Finally, misclassification rates were compared between pairs of blood tests using the McNemar test. Here, to simplify the presentation, the results are mainly expressed as the inverse proportion of correctly classified patients.

#### Statistical analysis

The diagnostic accuracy of each test was expressed as the area under the receiver operating characteristic (AUROC), the overall accuracy and detailed indices such as likelihood ratio and diagnostic odds ratio (15–17). AUROCs were compared using the non-parametric Delong test (18). Data were reported according to STARD statements (19) and thus analysed on an intention-to-diagnose basis. Importantly, there was no exclusion owing to false blood test results. The size of the population was that necessary to detect a significant difference between the two most accurate tests according to a preliminary study (11) in the diagnosis of cirrhosis, which was the diagnostic target with the least difference between blood tests. With an α-risk of 0.05, a β-risk of 0.2, cirrhosis prevalence of 0.11, AUROC correlation of 0.75 and bilateral testing, the required sample size was 910 patients for the following AUROC values: FibroMeter: 0.93, Fibrotest: 0.89, as provided by the previous study (11).

## Results

### Patient characteristics

The principal characteristics of the 1056 patients were as follows: sex: 59.5% male; mean age: 45.6±12.5 years; Metavir F stage: F0: 4.4%, F1: 43.5%, F2: 27.0%, F3: 14.0%, F4: 11.2%; and mean liver specimen length: 21±8 mm. Thus, the prevalence of the main diagnostic target, significant fibrosis, was 52%. There was no correlation between Metavir F stage and liver specimen length (*r*_s_: 0.00, *P*=0.872), thus reflecting the pathologist expertise (14); 84.0% of liver specimens were ≥15 mm and 58.2% were ≥20 mm.

### Overall performance

The main indices of the diagnostic accuracy for significant fibrosis of the tests are presented in Table 1. AUROC for different diagnostic targets are presented in Table 2. Briefly, AUROC values for cirrhosis were higher than those for severe fibrosis, which in turn were higher than those for significant fibrosis as the diagnostic target. The AUROC of FibroMeter was significantly higher than that of other blood tests for significant fibrosis, severe fibrosis and cirrhosis (with the exception of Hepascore in cirrhosis). Globally, AUROCs of other blood tests were not significantly different (exceptions: Fibrotest superior to Hepascore for significant fibrosis, Fib-4 superior to APRI for severe fibrosis and cirrhosis and Hepascore superior to APRI for cirrhosis). The plots of the diagnostic accuracy as a function of blood test values are depicted in Figure 1. They clearly show that accuracy was maximum at the extremes and minimum in the middle of blood test values.

**Table 2:** AUROC (95% CI) of blood tests (grey cells) and their comparisons (*P*-value of the Delong test) as a function of the diagnostic target

	Significant fibrosis	Severe fibrosis	Cirrhosis
FibroMeter	0.853 (0.830–0.876)	0.885 (0.863–0.906)	0.907 (0.885–0.929)
Fibrotest	0.811 (0.785–0.838)	0.837 (0.809–0.865)	0.882 (0.855–0.910)
Hepascore	0.784 (0.757–0.812)	0.834 (0.806–0.862)	0.896 (0.868–0.924)
APRI	0.786 (0.759–0.814)	0.822 (0.792–0.852)	0.841 (0.803–0.880)
Fib-4	0.799 (0.772–0.825)	0.843 (0.816–0.871)	0.869 (0.835–0.903)
All blood tests	**<10^−3^**	**<10^−3^**	**<10^−3^**
FibroMeter vs. Fibrotest	**<10^−3^**	**<10^−3^**	**0.041**
FibroMeter vs. Hepascore	**<10^−3^**	**<10^−3^**	0.203
FibroMeter vs. APRI	**<10^−3^**	**<10^−3^**	**<10^−3^**
FibroMeter vs. Fib-4	**<10^−3^**	**<10^−3^**	**0.008**
Fibrotest vs. Hepascore	**0.004**	0.307	0.592
Fibrotest vs. APRI	0.059	0.324	0.131
Fibrotest vs. Fib-4	0.232	0.863	0.605
Hepascore vs. APRI	0.860	0.479	**0.013**
Hepascore vs. Fib-4	0.327	0.587	0.202
APRI vs. Fib-4	0.210	**0.037**	**0.021**

Significant differences are in bold characters.

APRI, aspartate aminotransferase to platelet ratio index; AUROC, area under the receiver operating characteristic.

**Table 1:** Diagnostic indices of blood tests for significant fibrosis

Test	Cut-off*	κ†	Se	Spe	+PV	−PV	+LR	−LR	DOR	OA	AUROC‡
FibroMeter	0.419	0.560	80.0	76.0	78.5	77.6	3.33	0.26	12.65	78.1	0.853
Fibrotest	0.435	0.493	67.7	81.9	80.1	70.2	3.74	0.39	9.48	74.5	0.811
Hepascore	0.465	0.450	66.2	79.1	77.5	68.3	3.17	0.43	7.42	72.4	0.784
APRI	0.548	0.454	62.4	83.5	80.5	67.0	3.78	0.45	8.38	72.5	0.786
Fib-4	1.116	0.458	73.9	71.9	74.2	71.6	2.63	0.36	7.25	73.0	0.799

* Diagnostic cut-off determined *a posteriori* according to maximum Youden index; the maximum overall accuracy cut-off (not shown) was close owing to prevalence (0.52) of diagnostic target.

† κ index measuring the agreement between a blood test and a liver biopsy for the diagnosis of significant fibrosis.

‡ AUROC is independent of cut-off.

AUROC, area under the receiver operating characteristic; DOR, diagnostic odds ratio; LR, likelihood ratio; OA, overall accuracy; PV, predictive value; Se, sensitivity; Spe, specificity.

### Performance profile

**Fig. 2:**
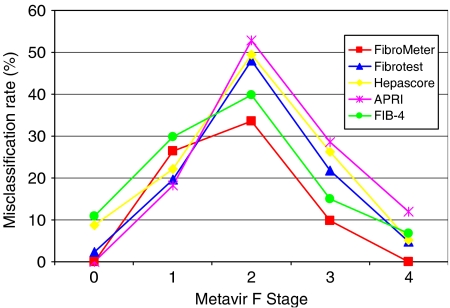
Misclassification rate of five blood tests for significant fibrosis as a function of Metavir fibrosis stages.

The misclassification rates of blood tests for significant fibrosis based on liver biopsy as a function of Metavir F stage are presented in Figure 2. The comparisons of correct classification rates are detailed in the Supplementary Material (Table S1). Briefly, the overall rate of correct classification was significantly higher with FibroMeter compared with all other blood tests, which in turn did not show significant differences between them. The correct classification rate of significant fibrosis by FibroMeter was significantly higher than that of other blood tests in all single or combined stages of significant fibrosis. Conversely, the correct classification rate for non-significant fibrosis (≤F1) was significantly inferior with FibroMeter compared with Fibrotest and APRI.

### Reliable diagnosis

#### Predictive values

**Fig. 3:**
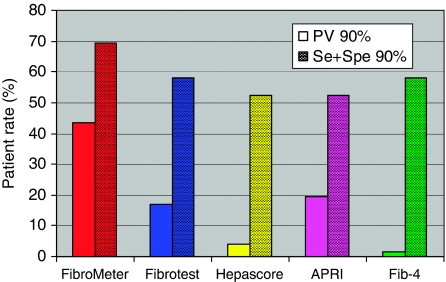
Patient rate (%) with reliable diagnoses defined either by ≥90% predictive values (PV) or by 90% Se/Spe for significant fibrosis according to five blood tests.

Predictive values can be easily inferred and compared from Figure 1. We first looked at traditional 90% positive predictive values (PPV) and negative (NPV) predictive values. The corresponding thresholds and patient rates are depicted in Figure 3 and detailed in the Supplementary Material (Table S2). Briefly, the proportion of patients with a reliable diagnosis for significant fibrosis by a blood test was in the following significantly decreasing order: FibroMeter: 43.5%, APRI: 19.6%, Fibrotest: 17.1%, Hepascore: 3.9% and Fib-4: 1.7% (*P*<10^−3^ between tests).

**Fig. 4:**
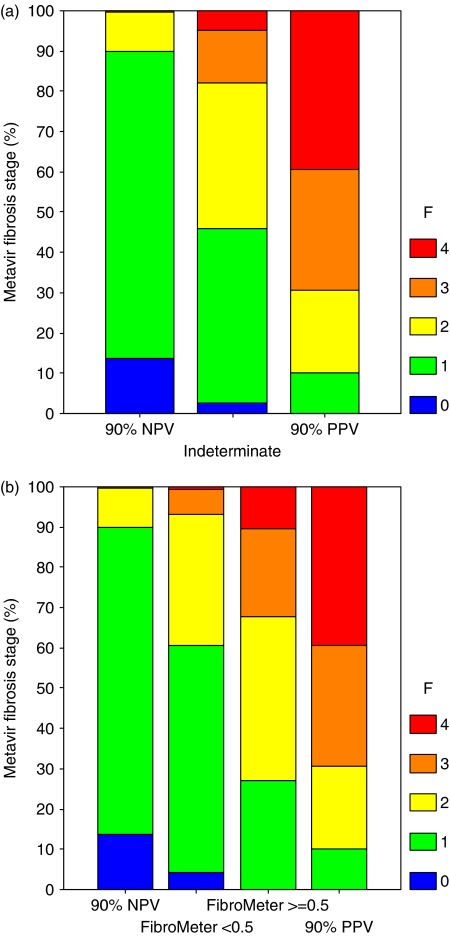
Proportions of Metavir fibrosis stage (F) according to thresholds of ≥90% negative (NPV) and positive predictive values (PPV) with a single indeterminate interval for significant fibrosis with FibroMeter (a). (b) The previous indeterminate interval was divided according to diagnostic cut-off at 0.5, providing two new intervals between this cut-off and the thresholds of predictive values: second interval between 90% NPV threshold and diagnostic cut-off; third interval between diagnostic cut-off and 90% PPV threshold.

Figure 4 shows the proportion of Metavir F stages as a function of reliable diagnosis intervals defined by the ≥90% PV for the most accurate test (FibroMeter). There was very little misclassification of extreme fibrosis stages in these two intervals; thus, there was no F0 in the FibroMeter interval with ≥90% PPV, whereas there was 0.5% of F3 and 0% of F4 in the FibroMeter interval with ≥90% NPV. In the indeterminate interval, 79.7% of patients were F1 or F2 and 17.8% of patients were F3 or F4 (Fig. 4a). However, when this indeterminate interval was divided into two new intervals according to the *a priori* diagnostic cut-off of 0.5 (Fig. 4b), in the interval between 90% NPV and <0.5 the proportion of F1 or F2 was 88.9% with 6.8% of severe fibrosis (F3+4) or 0.6% F4. The proportions in the interval between 0.5 and 90% PPV were: severe fibrosis (F3+4): 32.3%, F4: 10.5%, F1 or F2: 67.7% and F1/2/3: 89.5%. Thus, one can easily increase the reliable diagnosis rate by considering patients within the interval between ≥90% NPV threshold and <0.5 as F1 or F2. Consequently, we obtained three intervals with reliable diagnoses: F0 or F1 in the ≥90% NPV interval, F1 or F2 within the interval between 90% NPV threshold and <0.5 cut-off and ≥F2 in the ≥90% PPV interval (with a majority of F3 or F4: 69.2%). These three intervals with approximately 90% accuracy included 75.5% of the population, among whom 89.5% of the patients were correctly classified. It was possible to determine the same three reliable intervals with other blood tests (thresholds in Table S2). Finally, by including the new third interval (between 0.5 and 90% PPV), it was possible to obtain the following four intervals with the respective reliable diagnoses: F0/1, F1/2, F1/2/3 and F2/3/4 in 100% of the population, among whom 89.5% were correctly classified with FibroMeter.

We also looked at the robustness of PV thresholds. Indeed, PVs are usually defined by a threshold with a corresponding precise value, e.g. 90% NPV, but in fact PVs correspond to an interval of blood test values, e.g. with ≥90% NPV. The curves of NPV and PPV for the five blood tests are detailed in the appendix (Fig. S1). Briefly, NPV curves showed the following pattern of decreasing robustness for threshold, i.e. progressive decrease: FibroMeter, Fibrotest, Hepascore, Fib-4 and APRI, the latter three having non-robust (irregular) shapes for high values. The shapes of PPV remained roughly the same order but were more robust for thresholds.

#### Other indices

**Fig. 5:**
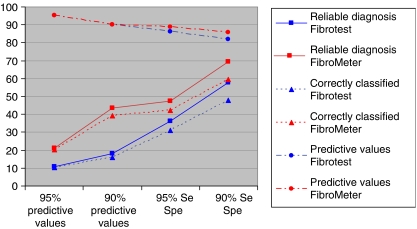
Rate (% of the whole population) on *y*-axis of patients with reliable diagnoses (squares), correctly classified patients (triangles) and resulting observed predictive values (PV) (circles) according to two intervals of reliable diagnosis defined by different predetermined thresholds (90 and 95%) of diagnostic indices (PVs, sensitivity (Se), specificity (Spe)) on *x*-axis for significant fibrosis by Fibrotest (blue) and FibroMeter (red).

**Fig. 6:**
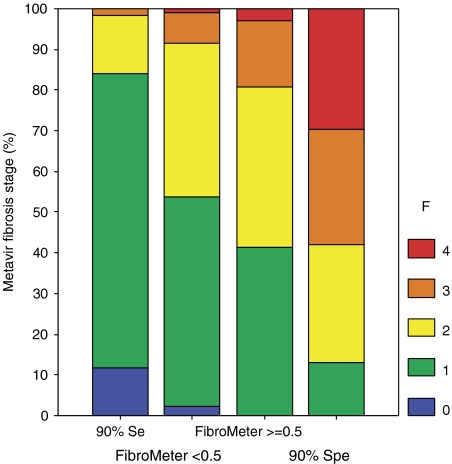
Proportions of Metavir fibrosis stage (F) according to 90% thresholds of Se/Spe for FibroMeter. The indeterminate interval between these two thresholds was divided according to diagnostic cut-off at 0.5, providing two new intervals between this cut-off and the thresholds of Se/Spe: second interval between 90% Se threshold and diagnostic cut-off; third interval between diagnostic cut-off and 90% Spe threshold.

In Table 3, we have determined other intervals of reliable diagnosis for the two most accurate tests by using other thresholds for PVs and sensitivity (Se) and specificity (Spe). Figure 5 shows that thresholds of 90% Se/Spe provided a higher patient rate with two intervals of reliable diagnosis (e.g. 69.5% for FibroMeter, gain: 26.0%) or correct classification at the expense of a minor loss in ensuing PVs (e.g. 4.3% for FibroMeter, Fig. 5) compared with the two intervals of reliable diagnosis defined by 90% PVs. Figure 6 shows the proportion of Metavir F stages as a function of reliable diagnosis intervals defined by ≥90% Se/Spe for the most accurate test (FibroMeter). There was no false extreme stage (F0 or F4) with 90% Se/Spe intervals in FibroMeter. When we divided the indeterminate interval according to the diagnostic cut-off, we observed in the new lower interval: severe fibrosis in 8.6% (F4: 1.0%) and F1 or F2 in 89.0%; and in the new upper interval: severe fibrosis in 19.2% (F4: 3.0%), F1 or F2 in 80.8% and F1/2/3 in 97%. The three intervals with reliable diagnosis (≤F1, F1/2, ≥F2) included 90.2% of the population, among whom 86.4% were correctly classified. Finally, by including the new third interval (between 0.5 and 90% Spe), it was possible to obtain the following four intervals with reliable diagnosis: F0/1, F1/2, F1/2/3, and F2/3/4 in 100% of the population, among whom 87.5% were correctly classified. Comparisons between reliable diagnoses provided by 90% PV or Se/Spe thresholds are presented in Table 4.

**Table 4:** Comparison of patient rates (%) with correct classification by FibroMeter in reliably diagnosed patients or in the whole population, as a function of 90% thresholds for predictive value or Se/Spe and the number of reliable intervals considered

Reliable intervals					Reliable diagnosis		(N)		Whole population	
					Patients (% whole population)		Correctly classified (%)		Correctly classified (%)	
*N*	F0/1	F1/2	F1/2/3	F2/3/4	PV	Se/Spe	PV	Se/Spe	PV	Se/Spe
2	x	–	–	x	43.5	69.5	90.0	85.6	39.2	59.5
3	x	x	–	x	75.5	90.2	89.5	86.4	67.6	78.0
4	x	x	x	x	100	100	89.5	87.5	89.5	87.5

Indeterminate intervals are in grey cells.

PV, predictive value; Se, sensitivity; Spe, specificity.

**Table 3:** Patient rates (% of the whole population) with reliable diagnoses defined by different thresholds of diagnostic indices for significant fibrosis by Fibrotest and FibroMeter

	Threshold	Patient rates with reliable diagnosis (%)		
Indices	(%)	FibroMeter	Fibrotest	*P*
Positive and negative predictive values	95	21.1	10.8	<10^−4^
	90	43.5	17.1	<10^−4^
Sensitivity and specificity	95	47.5	36.1	<10^−4^
	90	69.5	57.9	<10^−4^

### Sensitivity analysis

In order to test the influence of sensitive variables, performance was calculated in the whole population, then without the original population of the FibroMeter (8) and in liver specimen length ≥15 mm. Respective AUROCs were as follows: FibroMeter: 0.853, 0.847, 0.852; Fibrotest: 0.811, 0.810, 0.804; Hepascore: 0.784, 0.776, 0.775; APRI: 0.786, 0.790, 0.792; and Fib-4: 0.799, 0.798, 0.803 (*P*<10^−3^ between tests for each of the three comparisons). There was thus no change in the order of significance.

## Discussion

### Diagnostic accuracy

As Table 2 shows, the order of performance of blood tests for significant fibrosis was: FibroMeter > Fibrotest≈Hepascore≈APRI≈Fib-4. The present study validates the performance of the recently described simple test Fib-4 (6). However, this test was originally reported for the diagnosis of severe fibrosis where it was more accurate than APRI, the other simple test. In contrast, its accuracy was not significantly different from that of other tests for the usual diagnostic target of significant fibrosis, except for the FibroMeter. These differences in diagnostic characteristics are well explained by the performance profile. Indeed, FibroMeter had an appreciably higher accuracy rate than Fib-4 in all stages of significant fibrosis. Fib-4 was in turn more accurate than other tests (Fig. 2). The only test with 100% accuracy for significant fibrosis at the extreme fibrosis stages (F0 and F4) was FibroMeter.

Sensitivity analysis showed no particular effect of the original population on FibroMeter (8). The centre effect was examined in another study issued from the same cohort: the coefficient of variation of diagnostic cut-off of Fibrometer was low, 4%, and this was significantly lower than that of other blood tests (20). The long recruitment period and the proportion of patients not included (31.2%) are limitations of the study; therefore, an independent validation is desirable.

### Reliable diagnosis

Several cut-offs or thresholds can be distinguished within a diagnostic score or a blood test.

#### Diagnostic cut-off

The diagnostic cut-off distinguishes patients with or without the diagnostic target. Although diagnostic cut-offs are rarely reported, they are important and can be a source of discrepancy and/or controversy (21, 22). Because of this, determining and reporting the best diagnostic cut-off should always be done, especially in pivotal papers.

#### Predictive values

The other thresholds are those concerning a reliable diagnosis. A patient-based approach was adopted previously in this area, with thresholds corresponding to 90% PPV and NPV. In NPV and PPV ranges, a liver biopsy is considered to be unnecessary for fibrosis staging (3, 4). The plot of diagnostic accuracy as a function of blood test values (Fig. 1) shows the reliable diagnosis intervals determined by 90% PVs that varied markedly among tests, from 43.5% for FibroMeter to 1.7% for Fib-4 (Fig. 3).

However, these thresholds based on PVs have several limits.

Firstly, they are based on liver specimen as a reference, which has its own limits, i.e. sampling error (23) and observer variability (14). The inter-observer variability is high in intermediate fibrosis stages, especially Metavir F2 and F3 (14), and therefore could be largely responsible for the apparent decrease of performance of a blood test in intermediate fibrosis stages and corresponding blood test values. It could be argued that the variability of the blood test may be worse than the reference liver biopsy in intermediate stages. However, several lines of argument support the opposite. We have already observed that the variability of a blood test could be weaker than its reference. Thus, the dispersion of the area of fibrosis in each fibrosis stage was less when it was expressed as a blood test rather than the morphometric measurement from which it was derived (8). The blood test score is implemented in an original population with the less-biased measurements (specimen length, expert on consensus reading of liver biopsy). We have recently shown that the reproducibility of blood tests was far better than that of histological readings when blood tests and liver specimens were determined in routine conditions (24). Thus, the exportability of blood tests is easier and better than that of liver biopsy interpretation; in other words, it can be easier to reproduce a manufactured copy than the original hand-crafted manuscript.

Secondly, the PVs depend on the prevalence of the diagnostic target and are therefore not easily exportable. Thus, in the whole original population of Fibrotest (3), the 90% PV thresholds (corresponding Fibrotest values: 0.20 and 0.80) included 50% of the population vs. 17.1% in our population (corresponding Fibrotest values: 0.057 and 0.785). Then again, the previous Fibrotest threshold values of the original population (0.20 and 0.80) included 40.7% of patients in the present population (*P*<10^−4^ vs. 17.1%) with NPV at 81.0% and PPV at 91.8%. In the second group of the original Fibrotest population, the 90% PV thresholds corresponded to Fibrotest values of 0.10 and 0.60 (3). Thus, the differences in the reliable diagnosis rates are very significant between these three populations. This reflects the variability in PV, which is probably attributable to the difference in the prevalence of F0: 4% in the present population vs. 17% in the pivotal population of Fibrotest (3). In this respect, it would be more reliable to also report Se and Spe at a certain threshold because they do not depend on the prevalence of the diagnostic target. They can be used with confidence in studies on comparisons between populations. PVs can also be reported because they provide information on the diagnostic performance.

It should be kept in mind that Se/Spe reflects the diagnostic performance in a population whereas PVs reflect the diagnostic performance in a given patient. Because of this, clinicians prefer PVs. However, PVs are not adapted to blood tests because they are derived from carefully selected patients (hospital recruitment, liver biopsy performed) and thus difficult to use in patients close to the general population, i.e. in most clinical settings where blood tests are applied.

Thirdly, the thresholds of PVs are not always robust. Indeed, PVs, especially NPV, are very sensitive to the threshold with a non-progressive change in the high PVs, which is more marked in some tests. This has already been noted for Fibrotest (3), and is responsible for a variable PV for close blood test values and a marked variability of comparison between blood tests when the threshold varies, even slightly. All these causes of weak statistical robustness preclude reliable and extensive clinical application of PVs. It should be noted that thresholds of Se and Spe are robust because their plots as a function of blood test values are progressive by construction.

There are several possibilities to increase the reliable diagnosis intervals of blood tests.

#### Se/Spe

The first is to consider the intervals provided by Se/Spe indices as suggested previously. The present study (Fig. 5) shows that by using two (traditional) or three reliable intervals, it was possible to markedly increase the rate of patients correctly classified or reliably diagnosed with Se/Spe compared with PVs at the same 90% threshold level, at the expense of a weak loss of PV or accuracy.

#### New intervals defined by diagnostic cut-off

Secondly, as Figures 4 and 6 clearly show, in addition to the two traditional intervals of reliable diagnosis, one can delineate two additional intervals between the PVs or Se/Spe thresholds and the diagnostic cut-off. We thus obtain four successive intervals, allowing the following reliable diagnoses: F0/1, F1/2, F1/2/3 and F2/3/4. With FibroMeter, the rate of reliable diagnosis increased from 43.5 to 100% and that of the correct diagnosis from 39.2 to 89.5% (PVs) or 87.5% (Se/Spe). It is noteworthy that these figures of correct diagnosis correspond to the overall accuracy, which was thus markedly improved compared with the overall accuracy of the blood test initially designed for significant fibrosis (78.1%). The last two intervals included three fibrosis stages, which seems to be a broad diagnosis. However, the majority of patients were F0/1 in the first interval (90% considering the PV threshold), F1/2 in the two middle intervals (80%) and F3/4 in the last interval (69%).

#### What choice in clinical practice?

The first thing to consider is the number of reliable intervals. By increasing this number from 2 to 4, the rates of patients with reliable or correct diagnoses are increased (Table 4). The inconvenience is a change in the diagnostic target as the two middle intervals are not within the scope of the initial diagnostic target. However, the accuracy for fibrosis staging is increased. The second thing to consider is the type of thresholds. Se/Spe thresholds provide a marked increase in the rates of patients with reliable or correct diagnoses in the whole population at the expense of a small loss in accuracy among patients with reliable diagnoses compared with PVs (note that this loss decreases from 4.4 to 2% when increasing the number of reliable intervals, Table 4). However, this advantage is lost in the case of four reliable intervals. Thus, we propose to choose the option of four reliable intervals, defined by PV thresholds (and diagnostic cut-off), which provides the highest rate of correct diagnosis (89.5%) in the whole population. Nevertheless, this proposal will need validation in other populations. If we consider only the diagnostic target of significant fibrosis, the indeterminate intervals with unreliable diagnosis (F1/2 and F1/2/3) might lead to a liver biopsy. However, these indeterminate intervals are mainly hampered by liver interpretation whereas several indirect arguments suggest that blood tests can be used reliably, especially when they are applied to the general population, provided pre-analytical and analytical causes of variability are controlled (12). These indeterminate intervals can also be circumvented by transforming blood tests into semiquantitative variables with corresponding Metavir F stages (20) or reduced by sequential algorithms (25).

In conclusion, the present study shows that there is no parallelism between the rates of overall accuracy and reliable diagnosis of blood tests. These characteristics were significantly different among the evaluated blood tests. A third and fourth interval between the PVs or Se/Spe thresholds and diagnostic cut-off can substantially expand the proportion of patients with reliable diagnoses for fibrosis staging compared with the previously published method, and even more so for overall accuracy (up to 89.5%). Finally, in clinical practice, a reliable diagnosis can be made as a function of blood test values divided into four intervals: F0/1, F1/2, F1/2/3 and F2/3/4, with the extreme intervals corresponding to the initial diagnostic target.

## Supplementary material

The following supplementary material for this article is available online:

**Table S1.** Accurate classification rate for significant fibrosis (grey cells) by the different blood tests as a function of fibrosis stage according to Youden diagnostic cut-off (see Table 1). Significant differences are in bold characters. A rate of 97.7% in F4 means that 97.7% of patients with cirrhosis were correctly classified as having significant fibrosis.

**Table S2.** Patient rates with reliable diagnosis defined by thresholds of 90% negative (NPV) and positive (PPV) predictive values for significant fibrosis according to the blood tests. For example, the four reliable intervals for FibroMeter were defined according to the following thresholds: 0 to 0.192, >0.192 to <0.5, ≥0.5 to <0.853 and ≥0.853 to 1.

**Fig. S1.** Negative (top) and positive (bottom) PV curves as a function of blood test value. APRI and Fib-4 were standardized by binary logistic regression.



This material is available as part of the online article from: http://www.blackwell-synergy.com/doi/abs/10.1111/j.1478-3231.2008.01789.x (this link will take you to the article abstract).

Please note: Blackwell Publishing is not responsible for the content or functionality of any supplementary materials supplied by the authors. Any queries (other than missing material) should be directed to the corresponding author for the article.

## Abbreviations

APRI: aspartate aminotransferase to platelet ratio index
AST: aspartate aminotransferase
AUROC: area under the receiver operating characteristic
NPV: negative predictive value
PACA: Provence/Côte d'Azur (French administrative region)
PPV: positive predictive value
Se: sensitivity
Spe: specificity
