# Supplementary material for: Evaluating the accuracy and increasing the reliable diagnosis rate of blood tests for liver fibrosis in chronic hepatitis C
Source: Liver Int. 2008 Nov;28(10):1352–62. doi: 10.1111/j.1478-3231.2008.01789.x (PMC2711538; doi:10.1111/j.1478-3231.2008.01789.x)
Supplement: Supplementary file 1 [file liv0028-1352-SD1.doc]

Appendix for online supplement.

Table A. Accurate classification rate for significant fibrosis (grey cells) by the different blood tests as a function of fibrosis stage according to Youden diagnostic cut-off (see table 1). Significant differences are in bold characters. A rate of 97.7% in F4 means that 97.7% of patients with cirrhosis were correctly classified as having significant fibrosis.

|  | **Fibrosis stage** | | | | | | | | |
| --- | --- | --- | --- | --- | --- | --- | --- | --- | --- |
|  | **0** | **1** | **2** | **3** | **4** | **All** | **< 2** | **≥ 2** | **≥ 3** |
| **Overall accuracy :** |  |  |  |  |  |  |  |  |  |
| FibroMeter | 100.0 | 73.6 | 66.4 | 90.1 | 100.0 | 78.1 | 76.0 | 80.0 | 94.5 |
| Fibrotest | 97.7 | 80.3 | 52.0 | 78.2 | 95.1 | 74.6 | 81.9 | 67.7 | 85.3 |
| Hepascore | 91.3 | 77.9 | 50.5 | 73.8 | 94.9 | 72.4 | 79.1 | 66.2 | 83.2 |
| APRI | 100.0 | 81.8 | 47.2 | 71.4 | 88.0 | 72.5 | 83.5 | 62.4 | 78.8 |
| FIB-4 | 89.1 | 70.2 | 60.2 | 85.0 | 93.2 | 73.0 | 71.9 | 73.9 | 88.6 |
| **Comparison^a^:** |  |  |  |  |  |  |  |  |  |
| All tests | **0.021** | **< 10^-3^** | **< 10^-3^** | **< 10^-3^** | **0.010** | **0.001** | **< 10^-3^** | **< 10^-3^** | **< 10^-3^** |
| FibroMeter vs. Fibrotest | NA | **< 10^-3^** | **< 10^-3^** | **0.001** | NA | **0.028** | **< 10^-3^** | **< 10^-3^** | **< 10^-3^** |
| FibroMeter vs. Hepascore | NA | **0.037** | **< 10^-3^** | **< 10^-3^** | NA | **< 10^-3^** | 0.108 | **< 10^-3^** | **< 10^-3^** |
| FibroMeter vs. APRI | NA | **< 10^-3^** | **< 10^-3^** | **< 10^-3^** | NA | **< 10^-3^** | **< 10^-3^** | **< 10^-3^** | **< 10^-3^** |
| FibroMeter vs. FIB-4 | NA | 0.211 | **0.022** | 0.059 | NA | **< 10^-3^** | 0.084 | **< 10^-3^** | **0.002** |
| Fibrotest vs. Hepascore | 0.180 | 0.180 | 0.706 | 0.127 | 0.706 | **0.050** | 0.104 | 0.249 | 0.131 |
| Fibrotest vs. APRI | NA | 0.683 | 0.178 | 0.086 | 0.083 | 0.118 | 0.612 | **0.014** | **0.018** |
| Fibrotest vs. FIB-4 | **0.046** | **< 10^-3^** | **0.008** | 0.117 | 0.527 | 0.173 | **< 10^-3^** | **0.006** | 0.286 |
| Hepascore vs. APRI | NA | 0.089 | 0.366 | 0.446 | 0.059 | 0.953 | **0.043** | 0.082 | 0.096 |
| Hepascore vs. FIB-4 | 0.739 | **0.007** | **0.007** | **0.019** | 0.593 | 0.694 | **0.007** | **0.001** | 0.080 |
| APRI vs. FIB-4 | NA | **< 10^-3^** | **< 10^-3^** | **< 10^-3^** | 0.058 | 0.734 | **< 10^-3^** | **< 10^-3^** | **< 10^-3^** |

NA: not available due to impossible calculation with a cell containing 0 patient.

^a^ Comparison of accurately classified patients between blood tests by Cochran test (all) or McNemar test (pair)

Table B. Patient rates with reliable diagnosis defined by thresholds of 90% negative (NPV) and positive (PPV) predictive values for significant fibrosis according to the blood tests. For exemple, the four reliable intervals for FibroMeter were defined according to the following thresholds: 0 to 0.192, >0.192 to <0.5, ≥0.5 to <0.853, and ≥0.853 to 1.

|  | **90% NPV** | |  | **90% PPV** | |
| --- | --- | --- | --- | --- | --- |
|  | Threshold | Patients (%) |  | Threshold | Patients (%) |
| **Tests:** |  |  |  |  |  |
| FibroMeter | 0.192 | 21.7 |  | 0.853 | 21.8 |
| Fibrotest | 0.057 | 3.6 |  | 0.785 | 13.5 |
| Hepascore | 0.039 | 0.3 |  | 1 | 3.6 |
| APRI | 0.093 | 0.2 |  | 1.140 | 19.4 |
| FIB-4 | 0.295 | 1.3 |  | 11.499 | 0.4 |
| **Comparison^a^ :** |  |  |  |  |  |
| All tests | - | **<10^-3^** |  | - | **<10^-3^** |
| FibroMeter vs. Fibrotest | - | **<10^-3^** |  | - | **<10^-3^** |
| FibroMeter vs. Hepascore | - | **<10^-3^** |  | - | **<10^-3^** |
| FibroMeter vs. APRI | - | **<10^-3^** |  | - | 0.002 |
| FibroMeter vs. FIB-4 | - | **<10^-3^** |  | - | **<10^-3^** |
| Fibrotest vs. Hepascore | - | **<10^-3^** |  | - | **<10^-3^** |
| Fibrotest vs. APRI | - | **<10^-3^** |  | - | **<10^-3^** |
| Fibrotest vs. FIB-4 | - | **<10^-3^** |  | - | **<10^-3^** |
| Hepascore vs. APRI | - | 0.655 |  | - | **<10^-3^** |
| Hepascore vs. FIB-4 | - | **0.008** |  | - | **<10^-3^** |
| APRI vs. FIB-4 | - | **0.003** |  | - | **<10^-3^** |

^a^ Comparison between blood tests by Cochran test (all) or McNemar test (pair)

Figure A. Negative (top) and positive (bottom) predictive value curves as a function of blood test value. APRI and Fib-4 were standardized by binary logistic regression.
